# Heterogeneous Doping of Nanodiamond Grains with Exfoliated 2D NbSe_2_ Nanostructures for Highly Sensitive Ammonia Gas Sensors at Room Temperature

**DOI:** 10.1021/acsami.5c16578

**Published:** 2025-10-02

**Authors:** Adhimoorthy Saravanan, Bohr-Ran Huang, Deepa Kathiravan, Hsieh-Chih Tsai

**Affiliations:** † Graduate Institute of Electro-Optical Engineering and Department of Electronic and Computer Engineering, National Taiwan University of Science and Technology, Taipei 106, Taiwan; ‡ Graduate Institute of Applied Science and Technology, National Taiwan University of Science and Technology, Taipei 106, Taiwan; § Advanced Membrane Materials Center, National Taiwan University of Science and Technology, Taipei 106, Taiwan; ∥ R&D Center for Membrane Technology, Chung Yuan Christian University, Taoyuan 330, Taiwan

**Keywords:** semiconducting NbSe_2_, nanodiamond−NbSe_2_, nanodiamond NH_3_ sensors, NbSe_2_ ammonia detection, nanodiamond−NbSe_2_ band gap

## Abstract

Herein, a first-time report details the development of a heterogeneous nanodiamond (ND) grain-niobium diselenide (NbSe_2_) hybrid for room-temperature ammonia (NH_3_) gas sensing. Exfoliated NbSe_2_ nanorods, potentially formed via sonochemical exfoliation, exhibit semiconducting behavior with a band gap of 2.29 eV. The ND–NbSe_2_ hybrid demonstrates higher NH_3_ selectivity compared to pristine NbSe_2_ and ND. This hybrid achieves a significantly higher response of 11.3% with faster response and recovery times (81.2 and 70.6 s) than those of ND (5.9%) and NbSe_2_ (4.5%) at a lower concentration of 100 ppm. Also, the stability of the as-fabricated ND toward NH_3_ gas is exceptional when compared to that of NbSe_2_. This explains the level of influence of ND on the present ND–NbSe_2_ hybrid heterostructure. Moreover, the heterojunction formation with a change in the resistivity of the sample is involved in the sensing mechanism. This can be ascribed to the correlation of energy gaps between the ND grains (4.43 eV) and NbSe_2_ nanorods (2.29 eV), which promotes electron transportation from the conduction band of NbSe_2_ to ND at the applied voltage. In addition, the NbSe_2_–ND hybrids offer excellent stability for long-term gas detection. Furthermore, it is expected that this study will inspire the development of 2D-NbSe_2_–nanodiamond hybrid materials for advanced gas-sensing applications.

## Graphical abstract

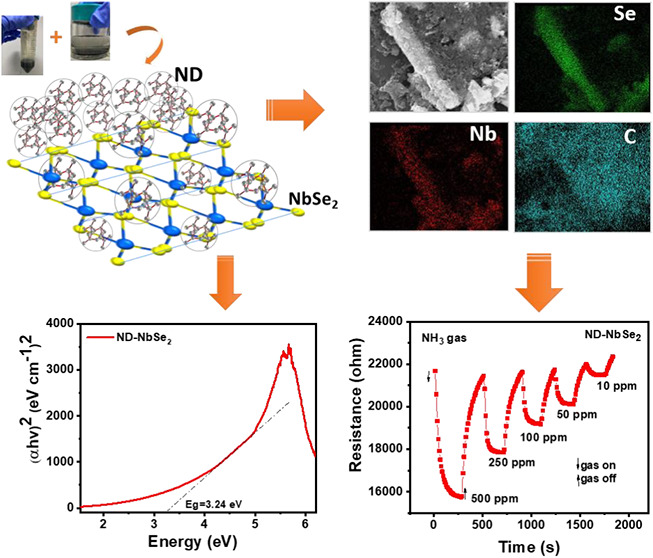

## 1 Introduction

Due to the increasing concern of global pollution, the search for effective and inexpensive materials for monitoring hazardous gases in the environment is extremely crucial. Ammonia (NH_3_) is one of the most frequently used chemicals.^1^ The detection of NH_3_ gas in day-to-day life is essential in terms of labor and environmental protection in chemical industries, food processing, and biomedical laboratories.^2^ The exposure limit of NH_3_ to humans is 35 ppm, suggesting that NH_3_ is really a precarious substance.^3^ The purpose of detecting low concentrations in the atmosphere is also compulsory to control air pollution with NH_3_.^4^ The exploration of effective and low-cost materials for gas sensors is particularly important for monitoring the concentrations of harmful gases in the air.

In this regard, high surface area and more active sites of two-dimensional (2D) transition metal chalcogenides (TMDs) make them excellent candidates for gas sensing. Recent research has explored deeper into the fascinating properties of NbSe_2_, mainly in its nanoscale form. In contrast to its metallic bulk structures,^5^ studies suggest that monolayer NbSe_2_ shows distinct semiconducting behavior. This transformation is attributed to a phenomenon called quantum confinement. In bulk NbSe_2_, the electrons can move freely throughout the material. However, quantum confinement controls electron movement within the atomically thin dimensions of a monolayer, leading to reformed energy levels and the formation of a band gap, a significant characteristic of semiconductors. The band gap in NbSe_2_ monolayers has been estimated to be around 1.2–1.8 eV, depending on the fabrication method. The semiconducting nature of NbSe_2_ monolayers makes them promising for nanoelectronics and allows for van der Waals heterostructures, expanding functionality.^6,7^

However, the integration of NbSe_2_ into carbonaceous materials (such as carbon nanotubes, nanodiamonds, graphene, and so on) is of great interest for achieving selective sensing properties toward NH_3_ gas.^8^ In particular, the use of nanodiamond with 2D TMDs has recently attracted considerable attention because of their high dispersion ability in low-boiling solvents.^9^ This ultradispersed nanodiamond shows resistance values of 0.217 MΩ and 5–10 nm in size. On the other hand, several exfoliation techniques, including thermal, mechanical, and chemical (liquid-phase exfoliation, LPE), can transform bulk transition metal dichalcogenides (TMDs) into layered structures.^10–12^ Among them, the LPE process employs sonication and centrifugation to generate a final dispersion of layered TMDs suitable for use in films or coatings.^9^

NbSe_2_ monolayers exhibit semiconducting behavior, making them attractive for gas-sensing applications. However, their selectivity can be limited. Here, we address this challenge by forming a hybrid material composed of NbSe_2_ and ND grains. In this context, this combination is envisioned to offer several advantages: (i) enhanced sensitivity due to the combined effects of NbSe_2_ (semiconducting response) and ND (excellent attraction for NH_3_ gas), and (ii) possibly enhanced selectivity through the particular contact between the NH_3_ gas molecules and diamond phase carbon band of ND grains. To the best of our knowledge, there are no reports on the hybridization of NbSe_2_ and nanodiamonds. Herein, we report for the first time the development of the heterogeneous structures for room-temperature (RT) NH_3_ gas sensing with excellent stability.

In this study, we report novel hybrid materials of ND grains and NbSe_2_ nanorods for NH_3_ gas-sensing applications. The illustration of nanorod formation was also elucidated in terms of the sonochemical synthesis. The present hybrid nanostructure shows enhanced sensitivity, stability, and selectivity toward NH_3_ gas detection at RT.

## 2 Experimental Section

### 2.1 Material Preparation

Niobium diselenide (NbSe_2_, 99.99%) powder was purchased from Alfa Aesar (Taiwan), and toluene was obtained from Sigma-Aldrich. ND powder (99.99%) was purchased from a supplier in China. The chemicals were used as received. Deionized (DI) water was used throughout the experiment. First, 2 wt % NbSe_2_ was dissolved in toluene solution for 25 h under ultrasound probe sonication (amplitude 2, 100 W, and a fixed scan speed was used) with continuous stirring with a time interval of 10 s every 5 min to avoid overheating of the samples. Subsequently, the sonicated solution was centrifuged at 5000 rpm for 20 min to remove the undispersed NbSe_2_ fragments or impurities. Second, 10 mg of ND powder was mixed in a 90% DI water/ethanol solution (10 mL) in an airtight container and continuously ultrasonicated for 25 h at room temperature in an ultrasonication water bath (40 kHz, 150 W). The supernatant solutions of both NbSe_2_ and ND were decanted separately into quartz bottles. For ND–NbSe_2_ preparation, 5 mL of NbSe_2_ was mixed with 5 mL of ND under vigorous stirring for 1 h. The samples were stored for thin film preparation and fabrication.

### 2.2 Characterization of Materials

The morphology and microstructures of the samples were studied using field emission scanning electron microscopy (FESEM, JSM-6500F) and transmission electron microscopy (TEM, Joel 2100F). Raman spectra were recorded using RENISAW inVia Raman microscopes (514.5 nm laser). X-ray photoelectron spectroscopy (XPS) was performed using a PHI Quantera spectrometer. AFM was performed using atomic force microscopy (Bruker Dimension ICON).

### 2.3 Fabrication and Sensor Testing

The ND–NbSe_2_ solution was drop-casted on a Si/SiO_2_ substrate and fabricated with an interdigitated Pd electrode pattern using radio frequency (RF) sputtering. Gas-sensing measurements were performed using a home-built gas-sensing system, which was connected to a mass flow controller (ppm) and computer-controlled power supply (2410). All gases were 99.99% pure and diluted in air. The time interval of periodically allowing and stopping the gases was set to obtain dynamic sensor response curves. N_2_ gas was used to break the vacuum of the gas-sensing chamber. A schematic representation of the preparation and fabrication of the sample is shown in Figure 1.

**Figure 1:**
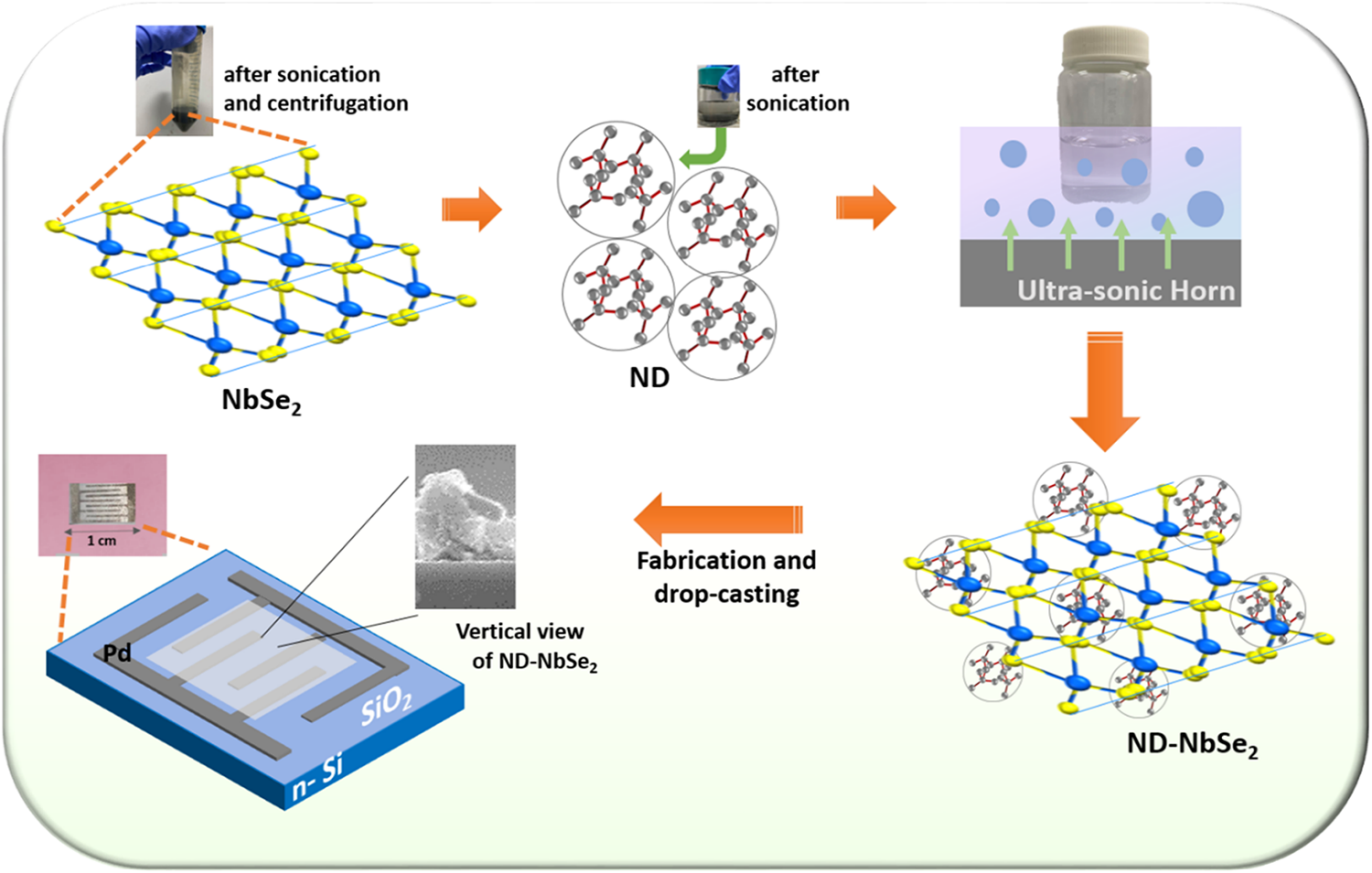
Schematic representation of the overall preparation and fabrication process of the ND–NbSe_2_ hybrid nanomaterial (insets show the photoimage of the as-fabricated device and FESEM cross-view image of ND–NbSe_2_).

## 3 Results and Discussion

The as-prepared NbSe_2_ and ND samples were first imaged using TEM, high-resolution TEM (HRTEM), and scanning TEM (STEM) mapping to study their structural properties with elemental analysis. The as-prepared samples were diluted to obtain clear images of TEM. Figure 2a,b[Fig fig2],c shows the TEM images of the NbSe_2_ and ND microstructures. The NbSe_2_ nanorod in Figure 2a appears like a core–shell structure, which is also confirmed through its STEM mapping, as shown in Figure 2b. Color mapping reveals the presence of elements (Nb, Se, N, and O) in the NbSe_2_ nanorods. Aside, a tiny sphere (is connected to each other) structure was observed for the ND sample with the honeycomb-like carbon lattices (inset of Figure 2c). In addition, the lattice spacing was also calculated from the HRTEM image of NbSe_2_ (Figure 2d), which was 0.63 nm with the (002) crystalline phase.

**Figure 2:**
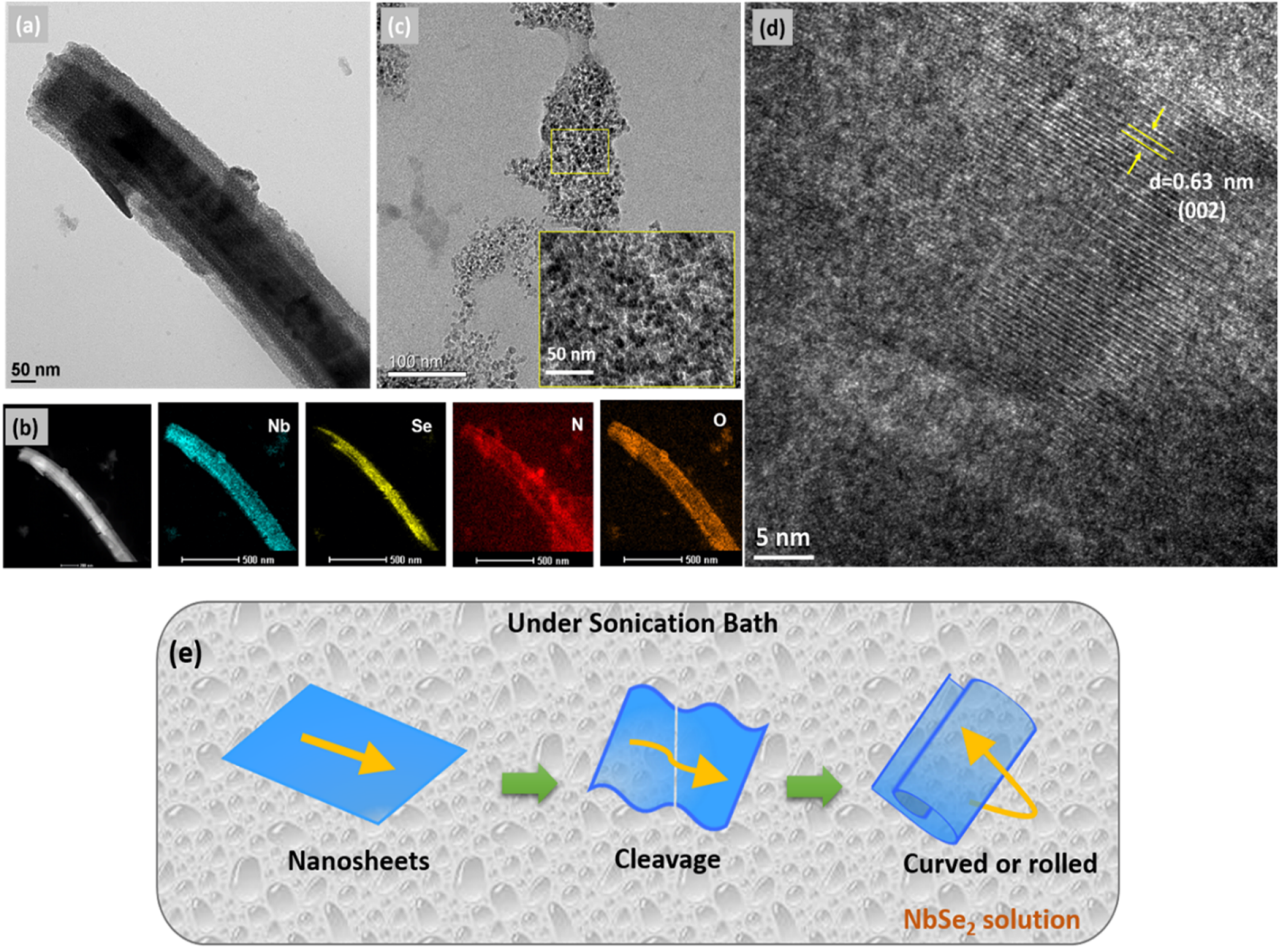
(a) TEM image of NbSe_2_ nanorods, (b) STEM elemental mapping of NbSe_2_, (c) TEM image of ND grains (the inset shows its HRTEM image), and (d) HRTEM image of NbSe_2_ nanorods. (e) Schematic representation of the rolled nanosheet structures of NbSe_2_ from its nanosheet structure.

Similarly, FESEM images of drop-cast NbSe_2_, ND, and ND–NbSe_2_ on Si/SiO_2_ substrates were also studied to determine the surface morphology of the samples. Figure 3a shows the NbSe_2_ nanorods, while Figure 3b exhibits a cluster of ND particles. Figure 3c shows the combined structure of the ND–NbSe_2_ hybrid, which is evenly distributed on the substrate. Additionally, FESEM-EDX and mapping were also performed to analyze the elemental composition of the present hybrid material. Figure 3d displays the FESEM-EDX with the inset showing the color mapping of different elements (i.e., Nb, Se, C, and O) that are presented in the ND–NbSe_2_ heterostructure.

**Figure 3:**
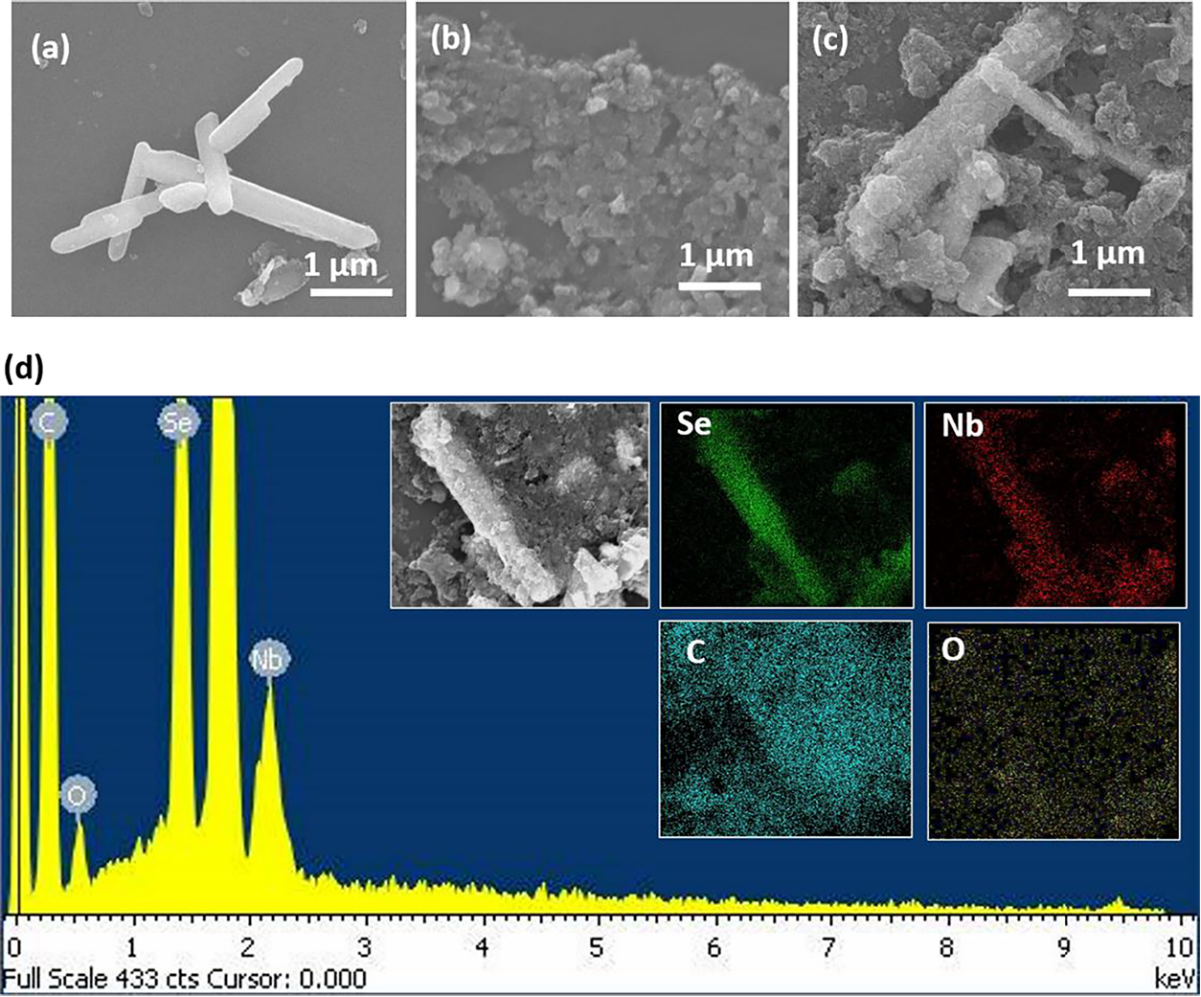
FESEM images of (a) NbSe_2_ nanorods, (b) nanodiamond grains, (c) ND–NbSe_2_ hybrid, (d) FESEM-EDX images of ND–NbSe_2_ hybrid (the inset shows its corresponding FESEM-elemental mapping).

The formation of nanorods can be ascribed to the sonochemical synthesis process that involves probe sonication. This probe delivers focused sound waves compared with an ultrasonic bath, producing a more localized and powerful effect. Continuous stirring is performed to confirm that the NbSe_2_ powder is evenly distributed throughout the solution. The nanorod formation involves high-frequency ultrasound waves from the probe sonicator inducing cavitation within the solution, leading to the formation and consequent intense breakdown of cavitation bubbles. This breakdown creates localized bursts of intense heat and pressure, stimulating the exfoliation of the NbSe_2_ precursor particles into smaller fragments. These fragments then serve as nuclei for the growth of NbSe_2_ nanorods under controlled conditions of sonication and stirring.

From the TEM and FESEM images, two possibilities were observed for the nanorod formation from the layered NbSe_2_ structure. Influenced by the intrinsic anisotropy of its layered structure, NbSe_2_ undergoes a transformation during sonochemical synthesis, potentially leading to the formation of two distinct nanorod morphologies (Figure 2e). First, the high-intensity ultrasound waves may induce exfoliative cleavage along specific crystallographic directions within the NbSe_2_ lattice. This preferential cleavage could result in the formation of aligned NbSe_2_ nanorods, where the longitudinal axis exhibits a high degree of alignment with the in-plane crystallographic directions of NbSe_2_. Second, the sonication process might promote curvature engineering, causing the exfoliated NbSe_2_ sheets to roll up into cylindrical nanostructures. These rolled-up structures essentially represent NbSe_2_ nanotubes with a closed geometry.

The ultraviolet–visible (UV–vis) spectra of NbSe_2_, ND, and ND–NbSe_2_ hybrid were obtained to further confirm their semiconducting behavior, as shown in Figure 4a–d. The corresponding Tauc plot was calculated for all of the samples using the UV–vis absorbance values. The Tauc plot method is a widely used method for assessing band gap energies of semiconductors. This method exploits the connection between the absorption coefficient (α) and incident photon energy (*h*υ) through the Tauc equation α*h*υ^*(n)*^ *= A*(*h*υ *– E*_g_), where *A* is a constant and *n* reflects the type of electronic transition (1/2 for direct, 2 for indirect). By plotting (α*h*υ)^(1/*n*)^ versus hυ and extrapolating the linear portion of the curve near the absorption edge to the *x*-axis, the band gap energy (*E*_g_) can be graphically determined (Figure 4b,c, and the inset of 4d). As is evident from the Tauc plots, the tangent lines yielded band gap values of 2.29 eV for NbSe_2_, 4.43 eV for ND, and 3.24 eV for the hybrid sample.

**Figure 4:**
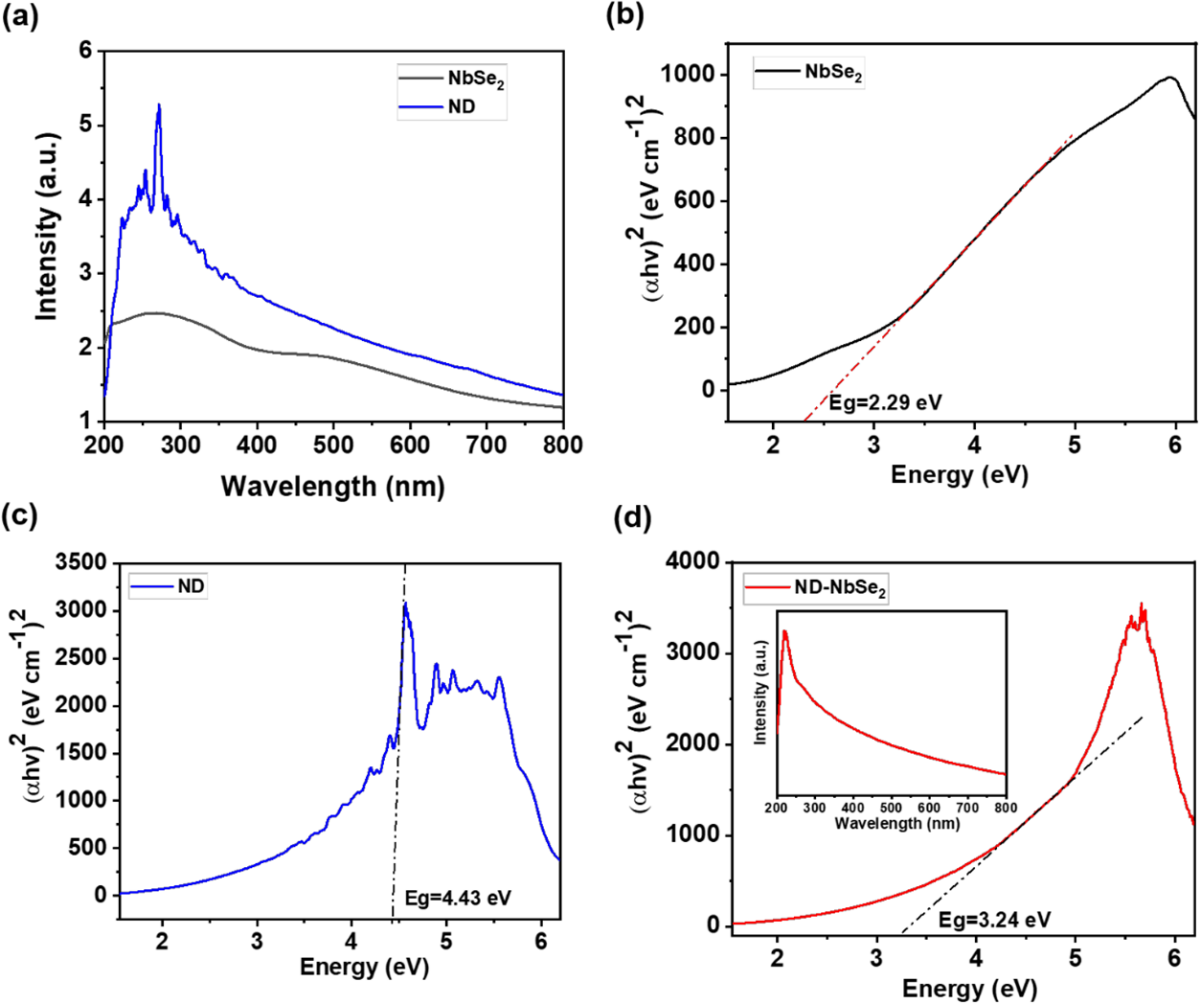
UV–visible absorbance spectra of NbSe_2_, ND, and the ND–NbSe_2_ hybrid and their corresponding Tauc plots.

Furthermore, AFM was performed to analyze the topologies of the samples. The overall structural arrangement and chemical composition of the ND–NbSe_2_ hybrid were discussed. However, the nanorod structure of NbSe_2_ is a significant part of this study. Thus, the size and height distributions of the nanorod structure before and after integration with ND particles should be investigated in detail. In particular, the single nanorod structures of NbSe_2_ and ND–NbSe_2_ were focused on using AFM and FESEM studies to analyze the correlation between ND and NbSe_2_. Figure 5a shows the AFM image of the NbSe_2_ nanorod with its height distribution (inset of Figure 5a), while the ND–NbSe_2_ nanorod is shown in Figure 5b. The nanospheres of the ND particles were covered with the NbSe_2_ nanorod when compared to the NbSe_2_ nanorod. It was also noted that the diameters of the NbSe_2_ and ND–NbSe_2_ samples were different, as shown in the insets of Figure 5a,5b. This can be attributed to the van der Waals interactions between NbSe_2_ and ND in the solution during the sonication process. Figure 5c,d reveals the depth histogram bar diagrams of the NbSe_2_ and ND–NbSe_2_ samples.

**Figure 5:**
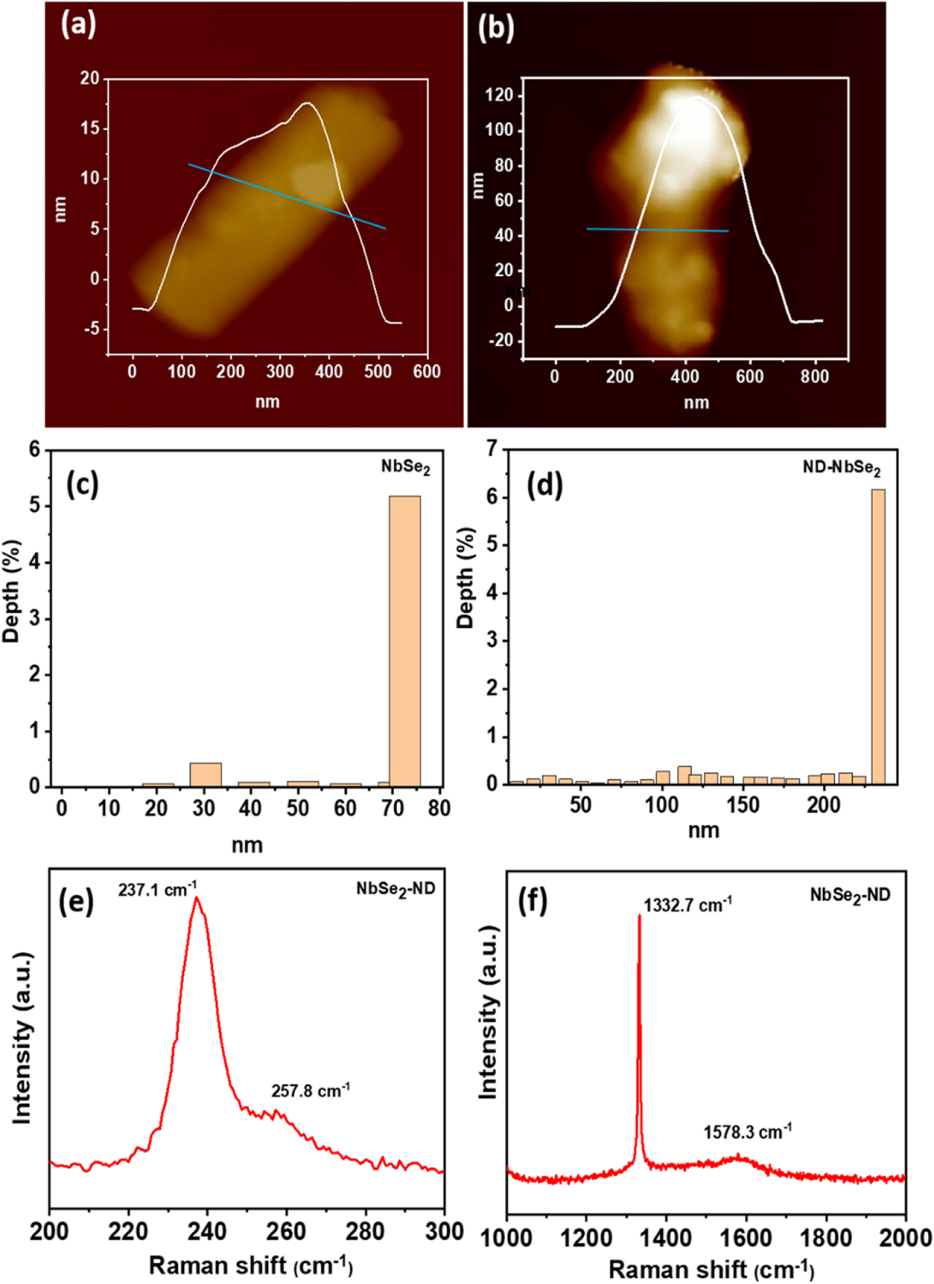
(a, b) AFM images showing the comparison of NbSe_2_ nanorods and NbSe_2_ nanorods with ND grains; the insets of (a, b) display their corresponding height and size distributions; (c, d) corresponding depth profile bar diagrams of NbSe_2_ nanorods and NbSe_2_ nanorods with ND grains. (e, f) Raman spectra of the ND–NbSe_2_ hybrid nanomaterial.

The Raman spectra of the drop-casted ND–NbSe_2_ heterostructure are shown in Figure 5e,f. Thus, ND–NbSe_2_ exhibits five peaks at 237.1, 267.8, 284.2,1332.7, and 1578.3 cm^–1^, which match those of previously reported NbSe_2_ nanorods and ND structures.^9,13,14^ Among them, the band at 237.1 cm^–1^ (Nb–Se, E_2g_^1^) justifies the active modes of the exfoliated NbSe_2_.^15,16^ The peak at 284 cm^–1^ indicates the out-of-plane soft modes of the NbSe_2_ nanorod structure.^17,18^ A sharp peak at 1332.7 cm^–1^ (D-band) and a small broad peak at 1598.3 cm^–1^ (G-band) are consistent with the Raman frequency of ND.^18,19^

High-resolution XPS spectra were used to study the stoichiometry of the hybrid nanomaterial, as shown in Figure 6. The binding energies observed at 202.3 and 205.2 eV in Figure 6a[Fig fig6],b are assigned to the niobium diselenide Nb 3d_5/2_ and Nb 3d_3/2_ orbitals with the Se 3d_5/2_ and Se 3d_3/2_ orbitals at 55.3 and 58 eV, respectively. These peaks are in good accordance with the oxidation states of Nb^4+^ and Se_2_ in NbSe_2_.^15^ Aside, the C 1s spectra (Figure 6c) of ND exhibit the C–C *sp* ^3^ peak at 284.5 eV, which can be attributed to the diamond phase, which is consistent with the Raman shift (at 1332.7 cm^–1^) of ND–NbSe_2_. Figure 6d shows the N 1s spectra at 394.2 eV, which are anticipated from the Nb–N and C–N bonds of ND–NbSe_2_. In addition, the binding energies of the O 1s peak (Figure 6e) were observed at 528.9 and 530.5 eV, which indicates the chemisorbed oxygen species and lattice oxygen to be expected from the ultrasonication process.^16,20^

**Figure 6:**
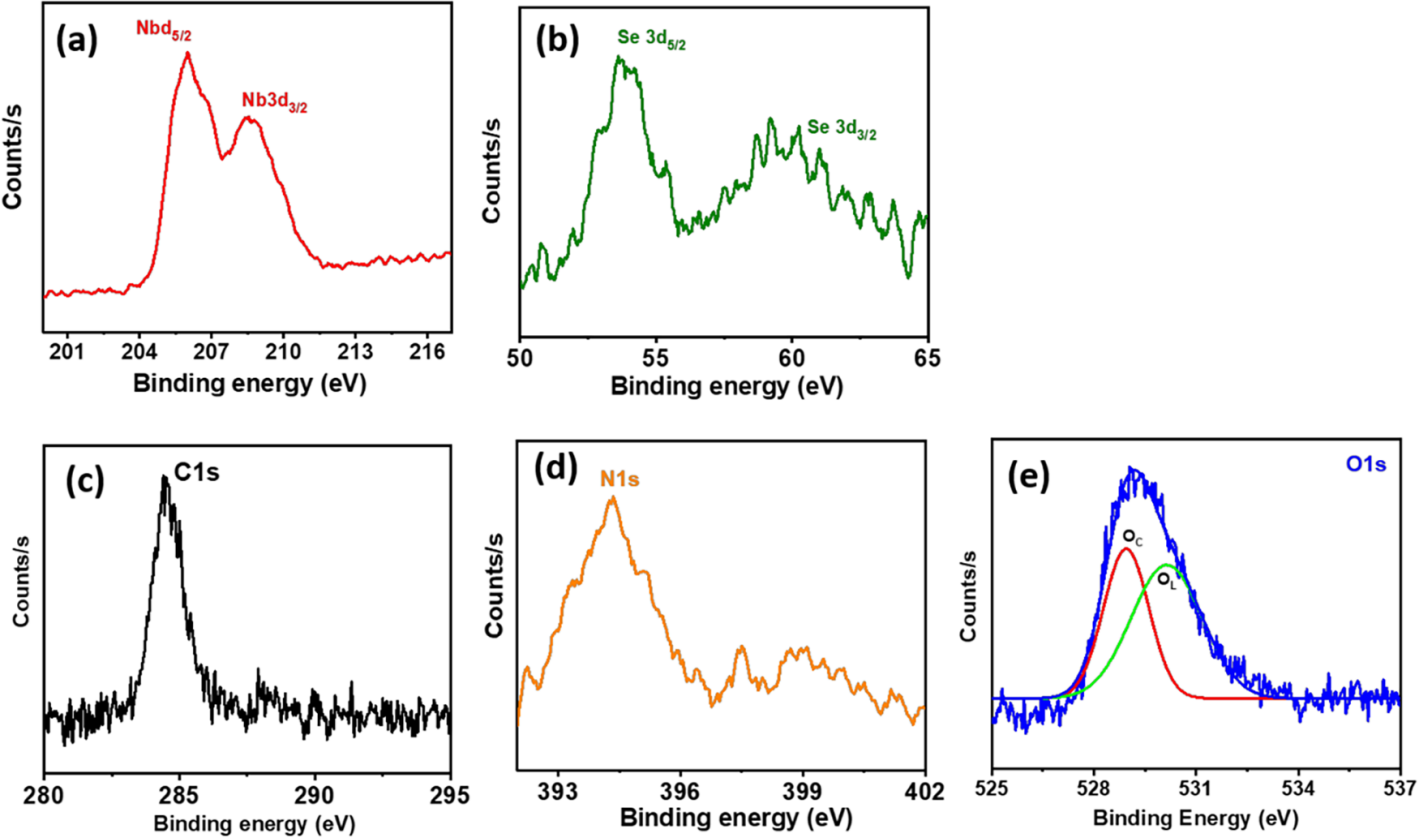
XPS spectra of (a) Nb 3d, (b) Se 3d, (c) C 1s, (d) N 1s, and (e) O 1s peaks of the ND–NbSe_2_ hybrid nanomaterials.

The gas-sensing properties of the as-fabricated NbSe_2_, ND, and ND–NbSe_2_ samples were investigated in a gas-sensing chamber with a mass flow controller by controlling different gas concentrations (ppm). The sensor response (%) from the change in the resistance was calculated using response (%) = *R*_g_/*R*_a_ × 100 for oxidizing gases and response (%) = *R*_a_/*R*_g_ × 100 for reducing gases, where *R*_a_ and *R*_g_ are the change in resistance in air and gas. Figure 7a shows the sensor response values of the as-fabricated NbSe_2_, ND, and ND–NbSe_2_ samples under 100 ppm of NH_3_ gas (RT of 26 *°*C, 35% RH). It was obviously revealed that the hybrid heterostructure of ND–NbSe_2_ exhibits a higher sensor response (11.3%) than those of ND (5.9%) and NbSe_2_ (4.5%), respectively. It should also be noted that ND exhibits good stability when compared to NbSe_2_, which stimulates the stable gas-sensing performances of the ND–NbSe_2_ hybrid.

**Figure 7:**
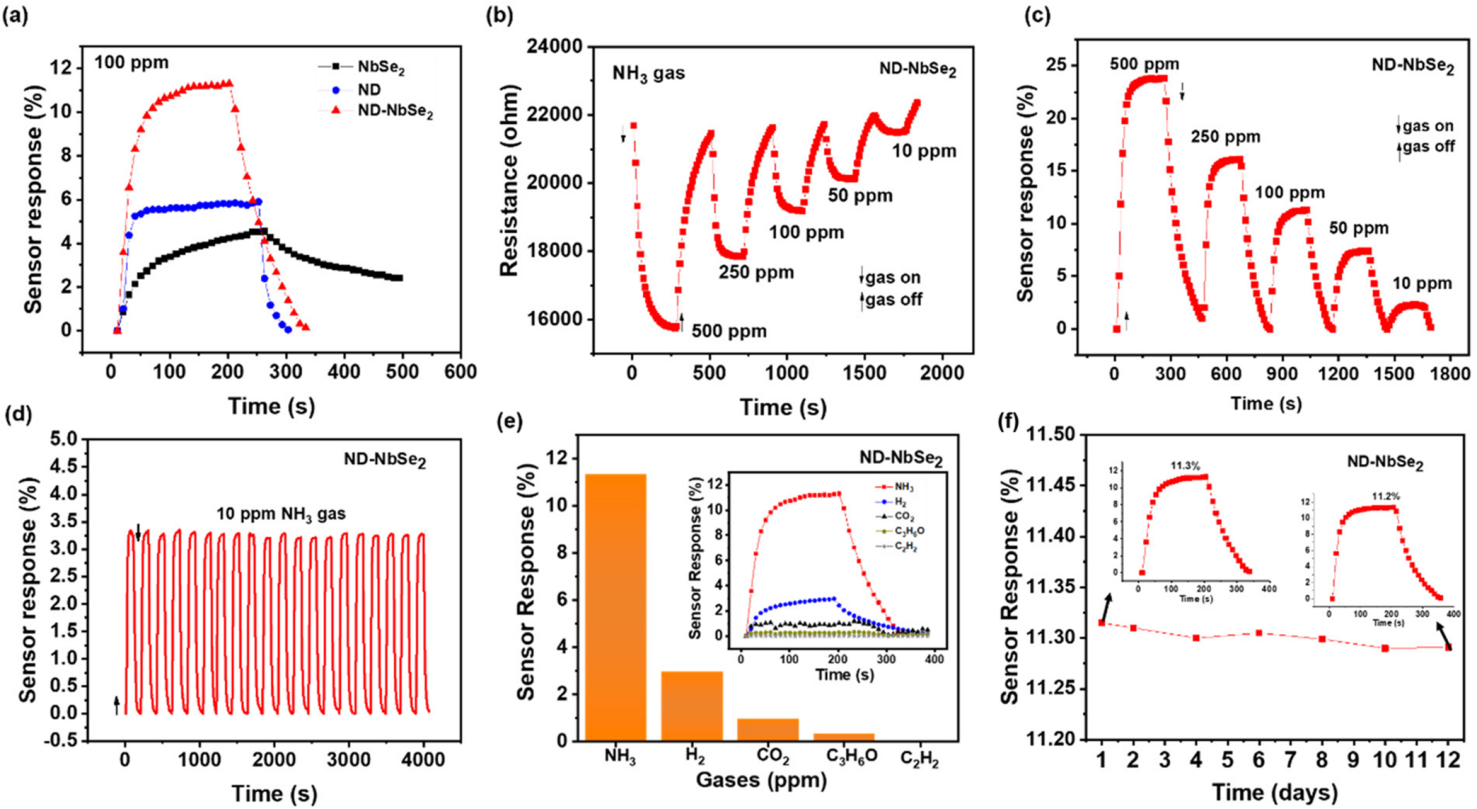
Gas-properties: (a) comparison of the sensor response of NbSe_2_, ND, and ND–NbSe_2_ at 100 ppm of NH_3_ gas, (b) transient sensor response curves with different NH_3_ gas concentrations (ppm), and (c) sensitivity plot. (d) Repeatability test with 20 cycles of 2 min on–off state, (e) selectivity test (inset shows the sensor response plot of different gases), and (f) long-term stability test for 12 days.

Figure 7b shows the dynamic transient curves of the change in the resistance of the as-fabricated ND–NbSe_2_ hybrid upon 5 min on–off state of various NH_3_ gas concentrations (500, 250,100, 50, and 10 ppm), and their corresponding gas sensor responses are shown in Figure 7c. The sensor response at 10 ppm is 3.3%, while a higher gas concentration of 500 ppm exhibits 24.8%. This illustrates the reliable performance of the present hybrid when switching between different gas concentrations. The repeatability of the present hybrid heterostructures is measured under 20 cycles of NH_3_ gas for a 2 min on–off state. Figure 7d shows the sensor response after 20 cycles of NH_3_ on–off state. Thus, the as-fabricated ND–NbSe_2_ hybrid heterostructure reveals exceptional repeatability even at 10 ppm, with each cycle exhibiting a sensor response of 3.3%. Furthermore, the selectivity test of the present hybrid was conducted at 100 ppm exposure to NH_3_, H_2_, CO_2_, C_3_H_6_O, and C_2_H_2_ gases. Figure 7e shows the sensor response of different gases using bar diagram and the inset of Figure 7e displays their corresponding sensor response/recovery times. The ND–NbSe_2_ hybrid consistently shows the strongest response to NH_3_ compared with other gases and confirming its selectivity. The long-term stability was measured for 12 continuous days to ensure the reliability of the present hybrid, as shown in Figure 7f.

In general, gas sensors at room temperature are affected by two important factors such as recovery time and the effect of relative humidity on the gas sensor response. In this work, a rapid recovery time was achieved using ND on the present hybrid, as shown in Figure 7a. The relative humidity was also measured under three conditions, namely, 35, 55, and 85% RH at RT of 26 *°*C under the exposure of 50 ppm of NH_3_ gas, as shown in Figure 8. The change in the RH values under 50 ppm NH_3_ gas exhibits a sensor response of 7.2%, suggesting that the present hybrid material is unaffected by RH. The present hybrid exhibits n-type semiconducting behavior during gas-sensing measurements. The interaction of gas molecules and ND–NbSe_2_ hybrid heterostructures is schematically shown in Figure 9a. Based on the energy band gaps of each nanomaterial, the electron transformation from NbSe_2_ to ND before and after exposure to the gas atmosphere is shown in Figure 9b. In the absence of gas exposure, the width of the potential barrier is expanded, which increases the resistance of the sensor and reduces its response. In contrast, the width of the potential barrier is reduced under NH_3_ gas exposure, which reduces the resistance of the sensor and enhances its response.

**Figure 8:**
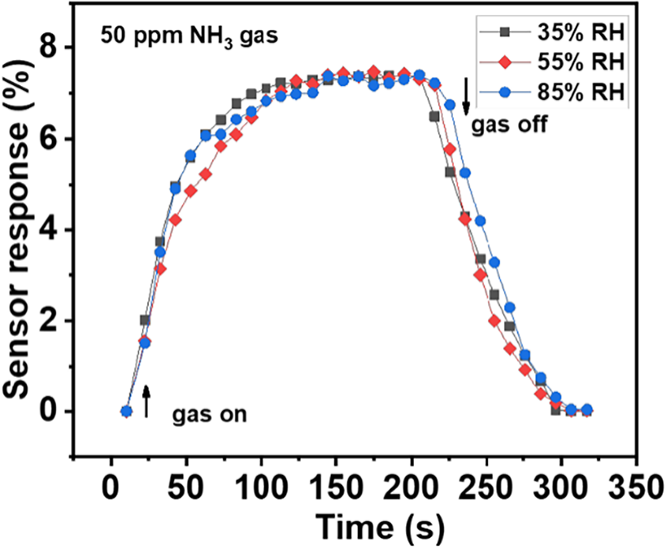
Relative humidity measurements of the ND–NbSe_2_ hybrid at 50 ppm NH_3_ gas.

**Figure 9:**
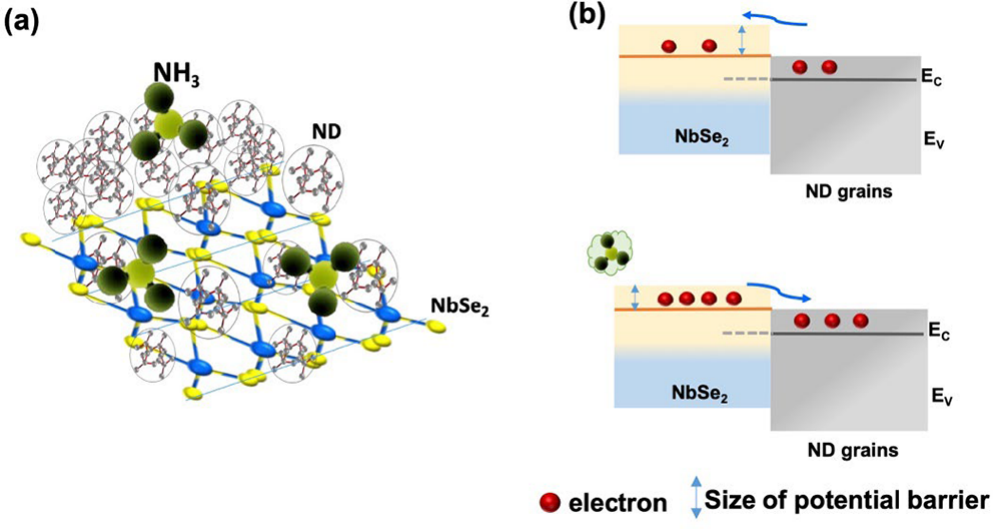
Schematic illustrations: (a) surface interaction of NH_3_ gas molecules with ND–NbSe_2_; and (b) the band model diagram of ND–NbSe_2_ under air and gas exposure.

The ND–NbSe_2_ hybrid exhibits n-type semiconducting behavior during NH_3_ sensing. The enhanced response arises from the complementary roles of ND and NbSe_2_, supported by spectroscopic, morphological, and sensing results. Role of NbSe_2_: NbSe_2_ nanorods serve as the charge-transport pathway. With a relatively narrow band gap of 2.29 eV (Tauc plot, Figure 4b), it enables efficient electron conduction, ensuring that variations in surface adsorption are rapidly transmitted. However, NbSe_2_ alone shows moderate sensitivity (Figure 7a), reflecting its limited adsorption capability. Role of ND: Nanodiamond grains, with a wide band gap of 4.43 eV (Figure 4c), contribute abundant adsorption sites, as confirmed by the sharp *sp* ^3^-carbon C 1s peak at 284.5 eV (Figure 6c).^21^ The lone-pair electrons of NH_3_ interact strongly with *sp* ^3^-hybridized carbon atoms, causing local charge redistribution and enhancing the sensing response. Nevertheless, ND alone is limited by its poor conductivity, leading to weaker sensitivity compared to the hybrid. Interface Role (ND/NbSe_2_ heterojunction): When combined, the hybrid shows an intermediate band gap of 3.24 eV (Figure 4d), consistent with the formation of a type-II heterojunction. TEM and FESEM images (Figures 2 and 3) confirm intimate ND–NbSe_2_ contact. At the interface, the band alignment produces a space-charge region that dynamically modulates under gas exposure. In air, oxygen adsorption (O 1s peak in XPS, Figure 6e) extracts electrons and widens the depletion barrier, increasing resistance. Under NH_3_ exposure, electron donation narrows the barrier and reduces resistance (Figure 9b). Synergistic Effect: The enhanced sensing behavior of the ND–NbSe_2_ hybrid results from the synergy of (i) efficient electron transport through NbSe_2_, (ii) abundant NH_3_ adsorption sites from ND, and (iii) space-charge modulation at the heterojunction interface. Comparative sensing tests (Table 1) confirm that the ND–NbSe_2_ hybrid outperforms ND and NbSe_2_ individually, substantiating the synergistic effect. Table 1 shows the comparison of present study to the ammonia gas sensors based on the 2D TMDs and carbonaceous hybrid nanomaterials.^22–33^ The combination of semiconducting NbSe_2_ nanorods (potentially influenced by quantum confinement), the n–n heterojunction, and the selective ND component likely leads to a synergistic effect, enhancing the overall response of the hybrid material towards NH_3_ gas detection with excellent stability.

**Table 1:** Comparison of NH_3_ Sensing Properties of Various 2D TMDs and Carbon Materials

2D-carbon hybrid materials	NH_3_ (ppm)	sensor response (%)	*T*_res_ (s)	*T*_rec_ (s)
nanodiamond^22^	100	5.2%		
functionalized GO^23^	100	12.2	60	80
PANI-MoS_2_ ^24^	5	10.9	98	57
rGO/SnO_2_ ^25^	300	4.7		
P–Si–MoS_2_ ^26^	100	2.2	22	30
Nanodiamond–CNT^27^	500	1.8	48	53
MXene/rGO fibers^28^	100	7.2		
P-doped graphene^29^	100	5.4	134	816
Diamond–MoS_2_ ^30^	100	0.18		
rGO–WS_2_ ^31^	10	121	60	300
Ar-MoSe_2_ ^32^	500	8.3	42	55
MoS_2_/WS_2_ composites^33^	5	∼7.5		
NbSe_2_^this study^	100	4.5	150	200
ND ^this study^	100	5.9	30	20
ND–NbSe_2_^this study^	**100**	**11.3**	**81**	**70**

## 4 Conclusions

In conclusion, we successfully report a facile approach for synthesizing NbSe_2_ nanorods and ND grains, enabling the subsequent fabrication of a well-defined hybrid material. Characterization techniques confirmed a higher distribution of ND grains compared with that of NbSe_2_ nanorods. Functionally, the hybrid material displayed n-type semiconducting behavior and a significant response to NH_3_ gas concentrations. Material analysis suggests an energy gap of 3.23 eV and the formation of an n–n heterojunction between the components, likely contributing to the sensing mechanism. Notably, XPS analysis revealed chemisorbed oxygen species that enhance the sensor response. Most importantly, the presence of ND’s diamond phase carbon band (C–C) with *sp* ^3^ hybridization is crucial for the exceptional NH_3_ gas-sensing properties. This work presents a promising strategy for the development of NbSe_2_-based hybrid materials with tailored functionalities for advanced gas-sensing applications.
